# Music in complex endoscopy: Effect of functional music on sedation requirements during endoscopic ultrasound and ERCP

**DOI:** 10.1055/a-2840-7071

**Published:** 2026-04-09

**Authors:** Volker Meves, Alexander Chamieh, Moustafa Mohamed, Hinrich Fehrendt, Christian Meinhardt, Alexander Arlt

**Affiliations:** 1 Gastroenterological Practice Syke, Syke, Germany; 2 37099 Gastroenterology, Klinikum Oldenburg AoR, Oldenburg, Germany; 3 139257 Internal Medicine, Israelitisches Krankenhaus Hamburg, Hamburg, Germany

**Keywords:** Pancreatobiliary (ERCP/PTCD), Endoscopic ultrasonography, Quality and logistical aspects, Sedation and monitoring, ERC topics, Intervention EUS

## Abstract

**Background and study aims:**

Previous studies in esophagogastroduodenoscopy or colonoscopy have demonstrated positive effects of music on patient anxiety and comfort. This study prospectively evaluated the effect of functional music on sedation requirements and satisfaction during complex endoscopic procedures.

**Patients and methods:**

A total of 144 patients undergoing endoscopic ultrasound or endoscopic retrograde cholangiopancreatography were prospectively randomized to either a functional music group or a control group without music. Sleep quality was assessed using the Pittsburgh Sleep Quality Index (PSQI) before the procedure. Sedation depth was monitored with bispectral index (BIS) monitoring. The primary endpoint was the amount of propofol required in mg/kg/min. Secondary endpoints included patient and staff satisfaction.

**Results:**

Propofol requirements did not differ significantly between groups (music: 0.15 ± 0.05 mg/kg/min; control: 0.17 ± 0.08 mg/kg/min, *P* = 0.77). Mean BIS values were similar (music: 60.2 ± 8.4 vs. control: 61.0 ± 8.4, *P* = 0.74). However, patient satisfaction was markedly higher in the music group (mean value 4.6 ± 0.6 vs. 3.9 ± 0.8, *P* < 0.00001), as was staff satisfaction (4.8 ± 0.4 vs. 4.1 ± 0.7, *P* < 0.00001). No adverse events through music intervention occurred. Analysis of PSQI subgroups revealed a trend toward lower propofol use in patients with impaired sleep quality (PSQI > 5) exposed to music, suggesting a possible modulatory effect in this subset.

**Conclusions:**

Functional music during complex endoscopic procedures did not reduce propofol consumption but significantly improved satisfaction for both patients and staff. The findings justify implementation of music as a non-pharmacological adjunct to improve patient comfort in endoscopic suites.

## Introduction

The influence of music on human sleep and relaxation has been documented since 1939 1 . Owing to its favorable cost-benefit ratio, use of music has been widely explored in medical settings, including anesthesia, intensive care, and endoscopy. Studies consistently report that music has a calming and anxiolytic effect without negative physiological consequences 2 3 4 5 . Furthermore, music impact on physiological parameters such as heart rate, blood pressure, and respiratory rhythm has been well-established 4 5 . In endoscopy, where environmental noises such as monitors and suction devices may induce arousal, background music can counteract stress responses by serving as environmental music therapy 6 7 . Most previous studies have relied on subjective measures of anxiety or sleep quality, such as the Pittsburgh Sleep Quality Index (PSQI). Few have incorporated objective parameters like electroencephalogram (EEG) or bispectral index (BIS) monitoring 8 . Functional or ambient music, as described by Bernardi et al., is characterized by slow, repetitive rhythms (40–80 Hz range) and absence of lyrics to avoid distraction 9 10 . Brian Eno’s Ambient 1: Music for Airports was selected as a representative composition for this study due to its proven relaxing character among patients with sleep disturbances [11,10].

Previous research has mainly investigated the effects of music during simpler procedures such as esophagogastroduodenoscopy and colonoscopy, frequently using benzodiazepine-based sedation 11 12 13 14 . Use of music during more complex procedures requiring propofol sedation, such as endoscopic ultrasound (EUS) or endoscopic retrograde cholangiopancreatography (ERCP), has not been sufficiently explored. Given the higher sedation depth required for such complex interventions, a measurable effect of music on sedative consumption was hypothesized. Therefore, the aim of this study was to analyze the effect of ambient music on sedation requirements and satisfaction in complex endoscopic procedures under nurse-administrated propofol sedation.

## Patients and methods

### Study design

A prospective, randomized, single-center study was conducted to evaluate the influence of functional music on propofol requirements during EUS and ERCP procedures at the University Hospital Oldenburg.

### Study population

All adult patients (≥ 18 years) who provided written informed consent and were scheduled for elective EUS or ERCP were eligible. Exclusion criteria included aged < 18 years, lack of consent, impaired decision-making capacity, impaired hearing or procedures requiring anesthesiologist-led sedation.

### Intervention

Participants were randomized to either a music or a control group. Functional ambient music by Brian Eno was played through a JBL Charge 5 speaker at a calibrated volume of 60 dB, measured with a Voltcraft SL-200 sound level meter. Music playback began before sedation and ended once the endoscope was withdrawn.

Sedation was administered using propofol, titrated to maintain a BIS value between 60 and 70. BIS values were recorded every 5 minutes. Patient satisfaction was assessed on a 5-point Likert scale (1 = unpleasant; 5 = very pleasant).

### Definition of complications

ERCP-related complications were predefined and assessed retrospectively based on clinical documentation and laboratory values in all patient records. The following definitions based on the ESGE recommendations were applied.

Post-ERCP pancreatitis (PEP) was defined as new or worsened abdominal pain after ERCP combined with an increase in serum lipase ≥ 3× the upper limit of normal measured after the procedure, in accordance with established consensus criteria.

Post-ERCP cholangitis was defined as postinterventional increase in inflammatory parameters (e.g. C-reactive protein and/or leukocyte count), with or without worsening cholestasis parameters, in the absence of another identifiable infectious source and requiring antibiotic therapy or clinical monitoring.

Minor bleeding was defined as intraprocedural bleeding that was immediately controlled endoscopically and did not require repeat endoscopy, radiologic or surgical intervention, or blood transfusion.

All patient charts were systematically reviewed for postprocedural symptoms, laboratory findings, and clinical follow-up according to these criteria.

### Data collection and analysis

Demographic and clinical data were collected pseudonymously. The primary endpoint was the weight-adjusted propofol dose (mg/kg/min). Secondary endpoints included satisfaction scores and team communication quality.

Statistical analysis was performed using IBM SPSS Statistics 27. Non-parametric tests (Mann-Whitney U) were used when normality assumptions (Shapiro-Wilk test) were not met. *P* < 0.05 was considered significant.

Sample size estimation was based on Rudin et al. 11 , who observed a 29.7% reduction in sedative use with music. Assuming a conservative effect size of 0.2 and power of 80%, the required sample size was 199 participants. An interim analysis after 6 months of recruiting was planned to assess efficacy, treatment effectiveness and to assess futility to save resources and protect participants.

### Ethics approval

The study was conducted in accordance with the standards set in the Declaration of Helsinki 1964 and its later amendments, and it was approved by the local ethic committee (Number 2022–008, University Oldenburg, Oldenburg, Germany).

## Results

At the time of the planned interim analysis after 6 months, a total of 144 patients were enrolled with 72 patients being randomized to the music group and 72 to the control group. In line with the study protocol the study was stopped due to clear results and to save resources and protect participants.

Baseline characteristics are summarized in Table 1 . Both groups were balanced in gender distribution (music 38 female/34 male vs. control 41 female/31 male; *P* = 0.64), body mass index (26.8 ± 4.1 kg/m² vs. 27.2 ± 4.5 kg/m²; *P* = 0.58), and type of procedure (EUS/ERCP; *P* = 0.77). The music group was older (64.1 ± 15.1 years vs. 58.6 ± 15.5 years; *P* = 0.0316) and exhibited a higher average PSQI (5.87 ± 3.92 vs. 4.59 ± 2.98; *P* = 0.0455), indicating a greater prevalence of sleep disturbance ( Table 2 ) as discussed in the discussion section.

**Table:** **Table 1** Baseline characteristics of the study population.

Parameter	Music group (n = 72)	Control group (n = 72)	*P* value
Age (years)	64.1 ± 15.1	58.6 ± 15.5	0.0316 ^*^
Gender (f/m)	38/34	41/31	0.64
BMI (kg/m²)	26.8 ± 4.1	27.2 ± 4.5	0.58
PSQI total score	5.87 ± 3.92	4.59 ± 2.98	0.0455 ^*^
Procedure (EUS/ERCP)	39/31	41/33	0.77
The two groups were well balanced in gender, BMI, and procedure type. Patients in the music group were slightly older and showed higher PSQI scores ( *P* < 0.05). BMI, body mass index; ERCP, endoscopic retrograde cholangiopancreatography; EUS, endoscopic ultrasound; PSQI, Pittsburgh Sleep Quality Index. ^*^ Statistically significant.			

**Table:** **Table 2** PSQI results.

Parameter	Music group	Control group
Total participants	74	74
Invalid questionnaires	0	4 (marked with "x")
Analyzable n	70	70
Minimum	0	0
Maximum	20	15
Mean	5.87	4.61
Median	5.0	4.0
Standard deviation	3.92	2.99
A total of 74 individuals participated in the control group, four of whom did not complete the PSQI questionnaire correctly (value "x"). Therefore, only the 70 valid datasets were included in the statistical analysis—matching the intervention group. A higher PSQI score indicates poorer sleep quality (cutoff: > 5 = poor sleeper). PSQI, Pittsburgh Sleep Quality Index.		

Table 3 provides an overview of indications for ERCP and EUS as well as corresponding complication rates in both study groups.

**Table:** **Table 3** Adverse events of procedures performed with vs. without music.

Procedure	Indication/parameter	Total	Music group	Control group	*P* value
ERC		46	22	24	1.000
	(Recurrent) choledocholithiasis	15	8	7	0.755
	Cholangioscopy for choledocholithiasis	3	2	1	0.600
	Cholangioscopy for stricture evaluation	5	2	3	1.000
	Stent exchange/removal (benign biliary stricture)	12	5	7	0.742
	Stent exchange/removal (malignant biliary stricture)	10	4	6	0.725
	Papillectomy	1	1	0	0.478
ERP		18	9	9	1.000
	Stent removal (benign pancreatic duct stricture)	12	7	5	0.620
	Advanced pancreatic duct therapy	6	2	4	0.620
	Pancreatoscopy for pancreatic duct stone	1	0	1	1.000
Papilla status					
	Native	4	4	0	0.050
	Prior endoscopic sphincterotomy	60	27	33	0.050
Adverse events ERCP	10%	6%	9%	1.000	
	Post-ERCP pancreatitis	2	1	1	1.000
	Post-ERCP cholangitis	2	1	1	1.000
	minor bleeding	2	1	1	1.000
EUS		80	39	41	1.000
	Diagnostic	68	31	37	0.220
	FNA/FNB	10	7	3	0.188
	EUS-guided drainage (LAMS)	2	1	1	1.000
Adverse events EUS	0	0	0	1.000	
Indications for ERCP and EUS as well as ERCP- and EUS-related adverse events are shown for both groups, including papilla status. Categorical variables are presented as counts and compared using two-sided Fisher’s exact test. *P* < 0.05 was considered statistically significant. ERC, endoscopic retrograde cholangiography; ERCP, endoscopic retrograde cholangiopancreatography; ERP, endoscopic retrograde pancreatography; EUS, endoscopic ultrasound; FNA, fine-needle aspiration; FNB, fine-needle biopsy; LAMS, lumen apposing metal stent.					

Overall, PEP occurred in one of 64 patients (1.6%). In line with the design of the study, focusing on non-emergency procedures, only four of 64 ERCP procedures (6.3%) were performed in patients with a native papilla, whereas the majority had undergone prior endoscopic sphincterotomy. Two cases of post-ERCP cholangitis were observed and were managed conservatively with antibiotic therapy. Minor intraprocedural bleeding occurred in two cases and was successfully treated endoscopically without need for repeat intervention, transfusion, or surgery. No further ERCP-related adverse events (AEs) were recorded. No EUS-related complications occurred.

### Sedation parameters

Average propofol consumption per kilogram body weight and minute did not differ significantly between groups (music 0.15 ± 0.05 mg/kg/min vs. control 0.17 ± 0.08 mg/kg/min; *P* = 0.77). Sedation depth was comparable, with mean BIS values of 60.2 ± 8.4 in the music group and 61.0 ± 8.4 in the control group ( *P* = 0.74). Procedure times were similar (39.2 ± 26.0 min vs. 53.5 ± 63.5 min; *P* = 0.30). Subgroup analysis of patients with poor sleep quality (PSQI > 5) suggested a trend toward lower propofol use in the music group (0.14 mg/kg/min vs. 0.17 mg/kg/min; *P* = 0.08), although this did not reach statistical significance.

### Satisfaction outcomes

Marked differences were observed in all satisfaction endpoints ( Table 4 ). Patient satisfaction scores were significantly higher in the music group (4.6 ± 0.6 vs. 3.9 ± 0.8; *P* < 0.00001). Similarly, satisfaction among assisting staff (4.8 ± 0.4 vs. 4.1 ± 0.7; *P* < 0.00001) and examiners (4.7 ± 0.5 vs. 4.2 ± 0.7; *P* < 0.00001) was significantly improved when background music was played. Subjective reports from staff suggested a calmer atmosphere and reduced stress during music exposure. Although all groups listened to the same piece of ambient music, satisfaction among assisting staff was slightly lower than that of patients or examiners, potentially reflecting repetition effects during multiple procedures.

**Table:** **Table 4** Sedation parameters and satisfaction ratings.

Variable	Music group	Control group	*P* value
Propofol dose (mg/kg/min)	0.15 ± 0.05	0.17 ± 0.08	0.77
BIS value (mean)	60.2 ± 8.4	61.0 ± 8.4	0.74
Patient satisfaction (1–5)	4.6 ± 0.6	3.9 ± 0.8	< 0.00001
Staff satisfaction (1–5)	4.8 ± 0.4	4.1 ± 0.7	< 0.00001
Examiner satisfaction (1–5)	4.7 ± 0.5	4.2 ± 0.7	< 0.00001
Although propofol dosage and BIS values did not differ significantly, satisfaction among patients, staff, and examiners was markedly higher in the music group (all *P* < 0.00001). BIS, bispectral index.			

### Safety of sedation

No sedation-related AEs or complications occurred. Oxygen saturation, heart rate, and blood pressure remained stable across both groups.

### Key results

**Fig. 1:**
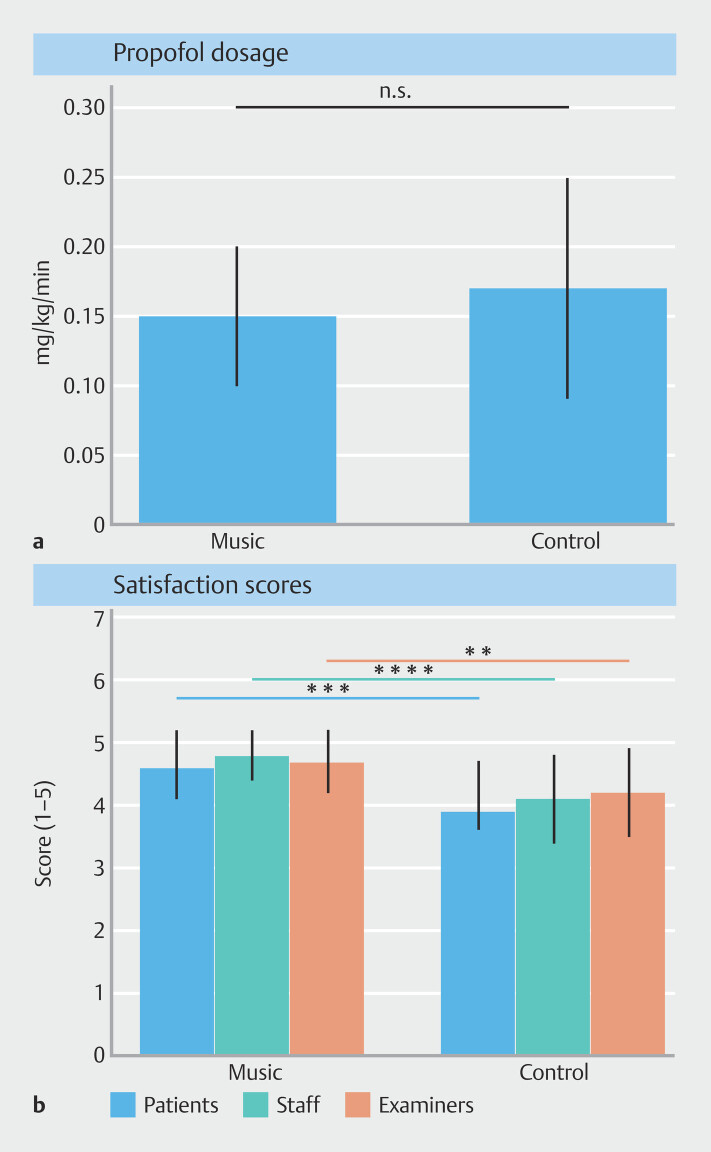
Propofol dosage and satisfaction score in patients undergoing complex endoscopic procedures. **a** Mean propofol consumption per kilogram body weight and minute (mean ± SD); no significant difference between groups (n.s.). **b** Satisfaction ratings (1–5) reported by patients, assisting staff, and examiners (mean ± SD). Significance is indicated separately for each category: black line and *** (patients, *P* < 0.001), dark grey line and **** (assisting staff, p < 0.0001), and light grey line and ** (examiners, *P* < 0.01). Each line connects the corresponding Music and Control groups. Error bars represent standard deviation.

Absence of a sedative-saving effect but substantial improvement in subjective satisfaction is illustrated in Fig. 1 . Panel **a** shows comparable propofol doses between groups (n.s.), whereas Panel **b** demonstrates significantly higher satisfaction scores across all participant categories.

## Discussion

Hospitalization and complex endoscopic interventions impose psychological stress on patients. Music, as a non-pharmacological adjunct, offers a simple, inexpensive, and side-effect-free option to alleviate this stress 15 .

### Physiological and sedative effects

The tempo and rhythmic structure of music influence autonomic responses 16 . Slow tempos (~60 bpm) synchronize with parasympathetic rhythms, promoting relaxation 17 . Functional ambient music was chosen to avoid personal biases associated with preference-based music and to ensure consistent exposure 18 .

No significant reduction in propofol dosage was observed, likely due to the deep sedation level (BIS ~60), where auditory perception and cortical responsiveness are suppressed 19 20 21 . Studies by Paspatis and DeWitt reported similar BIS targets for ERCP and EUS 22 23 . Nishiyama found maximal auditory evoked potentials at BIS 60, suggesting partial cortical processing persists 21 . However, short BIS sampling intervals may have missed transient responses.

Interestingly, patients with poor sleep quality (PSQI > 5) showed a trend toward lower propofol consumption, implying that preexisting autonomic instability could modulate music responsiveness—a finding warranting further exploration.

### Comparison with literature

Our findings align with those of Rudin 11 , Wang 24 , and Heo 22 , who found improved comfort and satisfaction but inconsistent sedative-saving effects. A meta-analysis by Wang et al. confirmed that music significantly reduces anxiety, heart rate, and systolic blood pressure, independent of musical genre or cultural context 24 .

In contrast, studies in lighter sedation (BIS 70–80) observed measurable sedative reduction 20 21 . This suggests that the effectiveness of music may depend on sedation depth and auditory processing thresholds.

### Environmental and team effects

Music may not only modulate patient anxiety but also affect the working environment. Staff reported reduced stress and improved concentration, consistent with findings from Canga et al. 6 , who demonstrated that environmental music therapy enhances team atmosphere and reduces acoustic stress.

### ERCP-related adverse events

The overall rate of ERCP-related AEs in our cohort was low. This finding should be interpreted in the context of the case mix. Notably and due to the design of the study (elective and non-emergency examinations), only a small proportion of procedures were performed in patients with a native papilla, whereas the vast majority had undergone prior endoscopic sphincterotomy. In addition, all procedures were performed following the European Society of Gastrointestinal Endoscopy recommendations for PEP prevention. Because native papilla cannulation is a well-established risk factor for PEP, the predominance of previously sphincterotomies in our cohort likely contributed to the low incidence of PEP observed. In addition, a substantial number of procedures consisted of stent exchange or removal, which are generally considered lower-risk interventions compared with primary biliary access. Nevertheless, given the limited sample size and retrospective design, absence of higher complication rates should be interpreted with caution and does not allow firm conclusions regarding procedural safety beyond the present cohort.

### Methodological limitations

Limitations include early study termination, a single musical composition, and fixed sound intensity. Room acoustics and absence of headphones may have influenced perception. In addition, the BIS interval (5 minutes) limited the ability to capture rapid fluctuations. Future studies should test multiple music types, variable timing (e.g. pre-procedural initiation), and continuous EEG monitoring to better capture subtle effects. A further important limitation concerns methodological aspects of trial design and execution. Randomization was performed without stratification, which resulted in baseline imbalances between groups, particularly with regard to age and PSQI scores. Given that impaired sleep quality may plausibly influence autonomic regulation and responsiveness to auditory stimuli, PSQI may have acted as a potential effect modifier. Although subgroup analyses suggested a trend toward lower propofol requirements in patients with PSQI > 5 exposed to music, these findings must be interpreted cautiously due to limited statistical power. Moreover, the originally planned sample size (n = 199) was not reached. The study was terminated after a prespecified interim analysis; however, early stopping inherently reduces statistical power and increases risk of type II error, particularly for the negative primary endpoint. Consequently, absence of a significant sedative-sparing effect cannot be interpreted as definitive evidence of lack of efficacy. Future adequately powered trials with stratified randomization are warranted to better delineate potential subgroup effects and to confirm the present findings.

### Clinical implications

Even without reducing propofol needs, the significant improvement in satisfaction highlights the clinical utility of background music. Its implementation requires minimal resources and aligns with European and German sedation guidelines 25 26 . Individual responsiveness—possibly linked to baseline anxiety or sleep patterns—should be explored further.

## Conclusions

Functional ambient music did not reduce sedative requirements during deep sedation but improved satisfaction for patients and staff. Given its simplicity, safety, and high acceptance, it should be incorporated into routine endoscopic practice. Future studies should address the timing, personalization, and neurophysiological mechanisms underlying its effects.
